# The Effect of Micronutrient Intake and Supplementation on Prevalent Diabetes‐Related Foot Ulcer and Incident Hospitalisation for Diabetes‐Related Foot Ulcer in Type 2 Diabetes: The Fremantle Diabetes Study Phase 2

**DOI:** 10.1002/jfa2.70181

**Published:** 2026-07-08

**Authors:** Emma J. Hamilton, Wendy A. Davis, Ranita Siru, Mendel Baba, Timothy M. E. Davis

**Affiliations:** ^1^ Medical School University of Western Australia Fremantle Western Australia Australia; ^2^ Endocrinology and Diabetes Department Fiona Stanley Fremantle Hospitals Group Fremantle Western Australia Australia; ^3^ Podiatry Department Sir Charles Gairdner Hospital Nedlands Western Australia Australia

## Abstract

**Objective:**

To determine whether micronutrient intake and/or supplementation is associated with prevalent diabetes‐related foot ulcer (DFU) and/or incident hospitalisation for DFU in type 2 diabetes.

**Research Design and Methods:**

Participants with type 2 diabetes from the community‐based Fremantle Diabetes Study Phase II (FDS2; 1499 participants, mean age 65.4 years, 51.8% males, recruited 2008–2011) were followed from entry to first hospitalization for/with DFU, death or 31 December 2016 (whichever came first). Cox proportional hazards modelling identified independent associates of prevalent DFU or first DFU hospitalization.

**Results:**

Self‐reported food frequency, including dietary intake of vitamin C, zinc and iron, taking vitamin supplements and B12 and iron levels were not independent associates of either prevalent DFU or incident DFU hospitalisation. Independent associates of prevalent DFU included younger age at diabetes diagnosis, being unmarried, retinopathy, distal symmetrical polyneuropathy (DSPN), previous diabetes‐related amputation, peripheral revascularisation or bypass. Incident DFU hospitalisation was independently associated with male sex, marital status, age at diagnosis of diabetes, HbA_1c_, a history of DFU, a history of peripheral revascularisation/bypass, heart rate, and ln(NT‐proBNP).

**Conclusions:**

Associates of prevalent and incident DFU included traditional risk factors such as DSPN and peripheral arterial disease. There was no association between nutritional status or taking vitamin supplements and prevalent DFU or incident DFU hospitalisation in people with type 2 diabetes.

## 1 Introduction

A diabetes‐related foot ulcer (DFU) is defined as a break of the skin of the foot (involving, as a minimum, the epidermis and part of the dermis) in a person with current or previously diagnosed diabetes which is typically accompanied by peripheral neuropathy and/or peripheral arterial disease in the lower extremity [1, 2]. DFU is a common complication of diabetes and is associated with a heavy burden of morbidity, disability, health care costs and death [3, 4, 5, 6]. Although there is evidence that some DFU related outcomes such as lower extremity amputation have improved in recent years, we have previously reported that DFU prevalence has not declined and incident DFU hospitalisations have increased in a community dwelling cohort of people with type 2 diabetes [7, 8, 9, 10]. It is critical to identify modifiable risk factors for DFU development in order to reduce the impacts of this highly burdensome condition.

There is growing interest in the relationship between nutrition and foot ulcer development and healing in people living with diabetes. The role of micronutrient supplementation in DFU healing was recently identified as being among the top 10 research priorities for diabetes‐related foot health and disease by Australian consumers with lived experience of diabetes and/or diabetes related foot disease (DFD), researchers and health professionals [11]. Hospital and specialist clinic based observational studies have found that a range of micronutrient deficiencies, such as of vitamin C, vitamin D, vitamin A, zinc, iron and selenium, are common in people with DFU and they have been associated with more severe disease in some studies [12, 13, 14]. Additionally, small interventional studies of micronutrient supplementation such as Vitamin C have shown promising improvements in DFU healing outcomes [14, 15]. There have, however, been no large cohort studies investigating the role of micronutrient intake in the development of DFU and/or DFU hospitalisation in people with type 2 diabetes.

The aim of this study was, therefore, to determine whether micronutrient intake (assessed by a food frequency questionnaire) and/or use of micronutrient supplementation was independently associated with either prevalent DFU or incident DFU hospitalisation in community dwelling people with type 2 diabetes.

## 2 Research Design and Methods

### 2.1 Study Site, Participants and Approvals

The Fremantle Diabetes Study Phase II (FDS2) is an observational, longitudinal study of people with known diabetes conducted in a zip code‐defined geographical area surrounding the port city of Fremantle in the state of Western Australia [16, 17]. There were approximately 157,000 people residing in the catchment area when FDS2 recruitment began in 2008. Socio‐economic data from this area at the time of FDS2 recruitment showed an average Index of Relative Socio‐economic Advantage and Disadvantage [18] of 1033 with a range by zip code of 977‐1113, figures comparable to the Australian national mean ± SD of 1000 ± 100.

The recruitment period for FDS2 was between 2008 and 2011, with follow‐up of diabetes complications and mortality data in the present study to end‐2016. Participants were identified from hospital, clinic and primary care patient lists, widespread advertising through local media, pharmacies, optometrists, networks of health care professionals, and third‐party mail‐outs to registrants of the Australian National Diabetes Services Scheme and the National Diabetes Register [17]. Details of recruitment, sample characteristics including classification of diabetes types, and non‐recruited patients have been published [17]. The FDS2 was approved by the Human Research Ethics Committee of the Southern Metropolitan Area Health Service (reference 07/397). All participants gave written informed consent prior to participation. In FDS2, 4639 people with diabetes were identified from the population, 1668 (36%) were recruited, and 1499 (90%) had clinically‐defined type 2 diabetes.

### 2.2 Baseline and Follow‐Up Assessments

Assessment at entry and at each second‐yearly face‐to‐face review included a comprehensive questionnaire, physical examination, and fasting biochemical tests performed in a single nationally accredited laboratory [17]. Details regarding medical conditions, demographic, socio‐economic and lifestyle data were collected. Dietary information was collected at study entry by use of an 88‐item, self‐administered, food frequency questionnaire, the Dietary Questionnaire for Epidemiological Studies Version 2 (DQESv2), Melbourne, a modification of the food frequency questionnaire that was developed by Cancer Council Victoria in the late 1980s to measure the dietary intake of people taking part in the Melbourne Collaborative Cohort Study [19]. Analysis of the DQESv2 for assessment of dietary intakes was undertaken by the Nutritional Assessment Office, Cancer Council Victoria, using nutritional information from NUTTAB 2010 and AUSNUT 2007, and the US Department of Agriculture database was used to calculate carotenoid intakes [9, 20, 21]. At the baseline face‐to‐face interview and subsequent biennial face‐to face interviews, participants were asked to bring all medicines they were currently taking (prescription, over‐the‐counter, and complementary/alternative therapies; including tablets, injections, inhalers, patches, and eye drops) and all available details were recorded, including use of vitamin and micronutrient supplements [22]. Comprehensive postal questionnaires which covered these data were sent to participants in the years between face‐to‐face assessments. The dose, quantity, frequency and duration of use of non‐prescribed medications were, however, not always documented and therefore have not been included in the present analyses.

Complications were identified using standard definitions [23] including albuminuria assessed by early morning spot urine albumin:creatinine ratio (uACR) measurement and renal impairment from the estimated glomerular filtration rate (eGFR) [24]. A general foot inspection was performed to detect the presence or absence of DFU located at or below the level of the malleoli, deformity, corns/callus, fissures, infections and nail pathology. Distal symmetrical polyneuropathy (DSPN) was defined using the clinical portion of the Michigan Neuropathy Screening Instrument [25, 26]. Patients were classified as having prevalent coronary heart disease (CHD) if there was a history of MI, angina, coronary artery bypass grafting, or angioplasty, and as having prevalent cerebrovascular disease if there was a history of stroke and/or transient ischaemic attack. Peripheral arterial disease (PAD) was defined as an ankle brachial index ≤ 0.90 or the presence of a diabetes‐related lower extremity amputation (LEA). A diabetes‐related lower extremity amputation included both minor and major lower limb resection.

In addition to usual care biochemical tests, vitamin B12 was measured using an E170 immunoassay analyser and reagents supplied by Roche Diagnostics (Castle Hill, NSW, Australia), and holotranscobalamin was measured using ‘Active B12’ reagents on an Alinity analyser (Abbott Diagnostics, North Ryde, NSW, Australia) in those with a serum total vitamin B12 < 250 pmol/L [27]. Serum N‐terminal pro‐brain natriuretic peptide (NT‐proBNP) was measured on an Elecsys 2010 (Roche Diagnostics Australia). Serum C‐reactive protein was measured on an Architect ci16200 analyser (Abbott Diagnostics Australia, North Ryde, NSW, Australia) using a high‐sensitivity protocol (hsCRP) with reagents supplied by Abbott Diagnostics. Participants were considered to have anaemia if they had a haemoglobin concentration ≤ 130 g/L in males and ≤ 120 g/L in females based on World Health Organisation criteria [28].

### 2.3 Ascertainment of Incident DFU Hospitalisation

Participants were followed to the first ever hospitalisation for/with DFU, death or end‐December 2016, whichever came first. The Hospital Morbidity Data Collection (HMDC) contains information regarding all public/private hospitalisations in WA since 1970, and the Death Register contains information on all deaths in WA [29]. FDS2 has been linked through the WA Data Linkage System to these databases, as approved by the WA Department of Health Human Research Ethics Committee, to provide validated data on incident events to end‐2016. Relevant International Classification of Disease (ICD)‐9‐CM (440.23 and 707.1 for lower extremity ulcer) and ICD‐10‐AM diagnosis codes (L97 and I70.23 for lower extremity ulcer, and E10.73, E11.73, E13.73 and E14.73 for foot ulcer) were used to identify incident DFU hospitalisation in the HMDC. Chart review was performed in available cases (all of those in the public sector) with ICD‐10‐AM codes L97 and I70.23, and, since ICD‐9‐CM codes did not include a specific diagnosis code for DFU, those with codes 440.23 and 707. The HMDC was also used to supplement data obtained through FDS assessments relating to prevalent/prior disease.

### 2.4 Statistical Analysis

The computer packages IBM SPSS Statistics 25 (IBM Corporation, Armonk, NY, USA) or Stata/SE 18.0 (StataCorp LP, College Station, TX, USA) were used for statistical analysis. Data are presented as proportions, mean ± SD, geometric mean (SD range), or, in the case of variables which did not conform to a normal or log‐normal distribution, median and inter‐quartile range [IQR]. Two‐sample comparisons were by Fisher's exact test for proportions, Student's *t*‐test for normally distributed variables, and Mann–Whitney *U*‐test for other variables. All clinically plausible variables with *p* < 0.200 in bivariable analyses and with < 2.5% missing data were considered for entry into the multivariable models. Multiple logistic regression identified independent associates of prevalent DFU. Backward elimination (conditional) was used with one variable manually removed at a time, the least significant first until all entered variables had *p* < 0.050. Cox proportional hazards modelling (backward conditional variable selection) was used to identify independent determinants of incident DFU hospitalisation during follow‐up. The proportional hazards assumption was tested for using time‐varying covariates and, when violated, adjusted for by adding the significant time‐varying covariates. A *p*‐value < 0.050 was considered statistically significant.

## 3 Results

### 3.1 Baseline Characteristics

The 1499 FDS2 participants with clinically‐defined type 2 diabetes and who lived in the postcode‐defined study area at recruitment were aged 65.4 ± 11.6 years at baseline, 51.9% were male, their median diabetes duration was 8.0 [2.5–15.3] years.

### 3.2 Prevalent Diabetes‐Related Foot Ulcer

Twenty‐two (1.5%) participants had an active DFU and another 22 had a history of hospitalisation for/with DFU before study entry. Table 1 summarises the baseline characteristics of those with versus without active or prior hospitalisation for/with DFU. The participants with a prevalent DFU and/or prior DFU hospitalisation were less likely to have an Anglo‐Celt ethnic background and were less likely to be married than those without a DFU. Mobility difficulties and need for assistance with activities of daily living were more common in those with prevalent/prior DFU. Participants with prevalent/prior DFU also had higher HbA_1c_, higher uACR, and higher rates of retinopathy, DSPN, nephropathy and macrovascular complications including heart disease and PAD. There was no difference in self‐reported dietary intake of vitamin C, zinc or iron and no difference in micronutrient supplementation (including Vitamin B12, Vitamin C, Vitamin D, zinc, and iron). Serum iron concentrations were lower in people with prevalent DFU, however there was no difference in serum ferritin.

**TABLE 1:** Baseline associates of prevalent (active or prior history) diabetes‐related foot ulcer (DFU) in FDS2 participants with clinically‐defined type 2 diabetes and living in the postcode‐defined study area at recruitment.

	*N*	No prevalent/prior DFU	Prevalent/prior DFU	*p*‐value
*N* (%)		1455 (97.1)	44 (2.9)	
Age (years)	1499	65.4 ± 11.6	65.5 ± 13.0	0.964
Sex (% male)	1499	52.0	50.0	0.879
Ethnic background (%)	1499			0.014
Anglo‐Celt		52.9	40.9	
Southern European		12.9	15.9	
Other European		7.4	< 11.4^a^	
Asian		4.4	< 11.4^a^	
Indigenous Australian		6.5	22.7	
Mixed/other		16.0	11.4	
Not fluent in English (%)	1499	10.8	11.4	0.807
Education (%)	1456			0.484
Primary or less		13.2	16.7	
Some/completed secondary		52.6	57.1	
Some/completed tertiary		34.2	26.2	
Married/de facto (%)	1499	63.5	43.2	0.007
Alcohol (standard drinks/day)	1435	0.1 [0–1.2]	0.1 [0–1.5]	0.931
Smoking status (%)	1489			0.174
Never		42.6	45.5	
Ex‐smoker		46.9	36.4	
Current smoker		10.5	18.2	
TV viewing ≥ 21 h/week (%)	1379	34.3	41.0	0.396
Any mobility problem (%)	1381	16.8	44.7	< 0.001
Any difficulty with ADL (%)	1394	6.5	25.6	< 0.001
Self‐reported food frequency				
Vitamin C intake (mg/day)	1353	98 (58–163)	95 (55–165)	0.779
Zinc intake (mg/day)	1353	11 (7–16)	9 (6–15)	0.141
Iron intake (mg/day)	1353	12 (8–18)	11 (7–17)	0.267
Taking vitamin B12 supplement (%)	1493	3.7	0	0.402
Taking vitamin C supplement (%)	1499	4.5	2.3	0.719
Taking vitamin D supplement (%)	1497	4.3	4.5	0.716
Taking zinc supplement (%)	1499	2.3	6.8	0.092
Taking iron supplement (%)	1494	2.3	2.3	> 0.999
Serum vitamin B12 (pmol/L)	1492	333 (210–527)	326 (189–561)	0.781
Serum active B12 (pmol/L)	360	48 (29–81)	42 (23–78)	0.438
Serum iron (μmol/L)	1491	16 (11–22)	14 (10–20)	0.026
Serum ferritin (μmol/L)	1460	117 (43–317)	119 (44–317)	0.943
Serum transferrin (μg/L)	1492	33 ± 5	32 ± 6	0.262
Anaemia (%)	1498	10.7	29.5	0.001
Age at diagnosis of diabetes (years)	1493	55.8 ± 12.2	48.3 ± 13.9	< 0.001
Duration of diabetes (years at study entry)	1493	8.0 [2.3–15.0]	15.5 [11.0–25.0]	< 0.001
Diabetes treatment (%)	1498			< 0.001
Diet		25.3	6.8	
Oral agents ± non‐insulin injectables		54.0	43.2	
Insulin ± oral agents ± non‐insulin injectables		20.7	50.0	
Fasting glucose (mmol/L)	1492	7.2 [6.2–8.8]	7.6 [6.2–10.4]	0.262
HbA_1c_ (%)	1494	6.8 [6.2–7.7]	7.4 [6.7–8.5]	0.006
HbA_1c_ (mmol/mol)	1494	51 [44–61]	57 [50–69]	0.006
Height (cm)	1496	166 ± 10	166 ± 11	0.827
BMI (kg/m^2^)	1494	31.3 ± 6.2	31.9 ± 5.3	0.488
Central obesity (% by waist circumference)	1489	70.9	81.8	0.130
Supine SBP (mm Hg)	1496	146 ± 22	149 ± 30	0.456
Supine DBP (mm Hg)	1496	80 ± 12	80 ± 12	0.905
Pulse pressure (mm Hg)	1496	65 ± 18	69 ± 23	0.315
Heart rate (bpm)	1492	70 ± 12	75 ± 13	0.009
On antihypertensive medication (%)	1494	73.1	77.3	0.608
Total serum cholesterol (mmol/L)	1494	4.4 ± 1.1	4.1 ± 1.2	0.128
Serum HDL‐cholesterol (mmol/L)	1494	1.24 ± 0.34	1.19 ± 0.36	0.379
Serum triglycerides (mmol/L)	1494	1.5 (0.9–2.5)	1.8 (1.0–3.2)	0.036
On lipid‐modifying medication (%)	1494	67.9	72.7	0.623
On aspirin (%)	1492	37.2	43.2	0.432
Serum hsCRP (mg/L)	1492	2.5 (0.8–7.6)	3.6 (0.9–11.9)	0.083
Urinary albumin:creatinine (mg/mmol)	1474	3.2 (0.8–11.9)	10.5 (1.7–65.7)	< 0.001
eGFR categories (%)	1494			0.014
≥ 90 mL/min/1.73 m^2^		39.3	27.3	
60–89 mL/min/1.73 m^2^		44.8	38.6	
45–59 mL/min/1.73 m^2^		8.6	15.9	
30–44 mL/min/1.73 m^2^		4.8	9.1	
< 30 mL/min/1.73 m^2^		2.6	9.1	
Serum NTpro‐BNP (pg/mL)	1492	77 (18–332)	173 (30–987)	< 0.001
Heart failure (%)	1499	6.1	18.2	0.006
Ischaemic heart disease (%)	1499	28.2	45.5	0.017
Atrial fibrillation (%)	1480	4.4	6.8	0.443
Cerebrovascular disease (%)	1499	7.9	27.3	< 0.001
Self‐reported claudication (%)	1499	8.7	27.3	< 0.001
Peripheral arterial disease (%)	1499	21.9	50.0	< 0.001
Distal symmetrical polyneuropathy (%)	1471	57.4	88.4	< 0.001
Diabetes‐related lower extremity amputation (%)	1499	0.1	20.5	< 0.001
Peripheral revascularisation/bypass (%)	1499	2.1	34.1	< 0.001
Any retinopathy (%)	1470	35.2	70.0	< 0.001
Depression (%)				
Any	1394	11.8	33.3	0.001
Major	1396	6.0	16.7	0.022

^a^ Small cell counts have been suppressed when there is the slightest possibility of identification.

The independent associates of prevalent DFU are shown in Table 2. These comprised younger age at diabetes diagnosis, being married (protective), retinopathy, DSPN, previous diabetes‐related amputation, and peripheral revascularisation or bypass. Micronutrient dietary intake and supplementation were not independently associated with prevalent DFU.

**TABLE 2:** Independent associates of prevalent diabetes‐related foot ulcer (DFU) in FDS2 participants with clinically‐defined type 2 diabetes (final model 40 DFUs/1466 [97.8%] participants).

Baseline variable	OR (95% CI)	*p*‐value
Married/de facto	0.34 (0.16, 0.73)	0.005
Age at diabetes diagnosis (increase of 10 years)	0.72 (0.55, 0.94)	0.017
Any retinopathy	2.51 (1.14, 5.50)	0.022
Distal symmetrical polyneuropathy	5.87 (1.98, 17.4)	0.001
Diabetes‐related amputation	20.9 (2.96, 147)	0.002
Peripheral revascularisation/bypass	12.0 (4.30, 33.3)	< 0.001

### 3.3 Incident Diabetes‐Related Foot Ulcer Hospitalisation

During 9831 person‐years (6.6 ± 1.9 years) of follow‐up to first incident DFU hospitalisation, death or end‐December 2016, whichever came first, 62 (4.1%) participants had an incident DFU hospitalisation, an incident rate (IR) of 6.3 (95% CI: 4.8, 8.1) per 1000 person‐years. Excluding the 44 with a prior or prevalent DFU, 44 (3.0%) had a first ever incident DFU hospitalisation, an IR of 4.6 (3.3, 6.1) per 1000 person‐years.

Baseline bivariable analyses for the type 2 diabetes cohort by incident DFU hospitalisation status are summarised in Table 3. Participants who had an incident DFU hospitalisation during follow‐up were more likely to have an Australian Aboriginal ethnic background and mobility problems, were less likely to be married, consumed more alcohol, had a younger age at diabetes diagnosis, longer diabetes duration, higher fasting serum glucose and HbA_1c_ despite more intensive hyperglycaemic management, had higher systolic blood pressure, pulse pressure and heart rate, higher ln(uACR), ln(hsCRP) and ln(NTproBNP), lower eGFR, and were more likely to have an active DFU, intermittent claudication, peripheral arterial disease, DSPN, diabetes‐related lower extremity amputation, and atrial fibrillation, a history of DFU, peripheral revascularisation/bypass, heart failure, and cerebrovascular disease (all *p* ≤ 0.041).

**TABLE 3:** Baseline associates of incident diabetes‐related foot ulcer hospitalisation in 1499 FDS2 participants with clinically‐defined type 2 diabetes and living in the postcode‐defined study area at recruitment.

	*N*	No incident diabetic foot ulcer	Incident diabetic foot ulcer	*p*‐value
*N* (%)		1437 (95.9)	62 (4.1)	
Age (years)	1499	65.4 ± 11.5	64.7 ± 15.1	0.721
Sex (% male)	1499	51.6	58.1	0.364
Ethnic background (%)	1499			0.010
Anglo‐Celt		52.7	46.8	
Southern European		13.1	9.7	
Other European		7.4	< 8.1^a^	
Asian		4.3	< 8.1^a^	
Indigenous Australian		6.4	21.0	
Mixed/other		16.0	12.9	
Not fluent in English (%)	1499	10.9	8.1	0.675
Education (%)	1456			0.643
Primary or less		13.1	5.0	
Some/completed secondary		52.9	50.0	
Some/completed tertiary		34.0	32.8	
Married/de facto (%)	1499	63.9	40.3	< 0.001
Alcohol (standard drinks/day)	1435	0.1 [0–1.2]	0.7 [0–1.8]	0.011
Smoking status (%)	1489			0.084
Never		43.0	35.5	
Ex‐smoker		46.6	45.2	
Current smoker		10.4	19.4	
TV viewing ≥ 21 h/week (%)	1379	34.1	43.6	0.150
Any mobility problem (%)	1381	16.3	44.8	< 0.001
Any difficulty with ADL (%)	1394	6.7	14.0	0.056
Self‐reported food frequency				
Vitamin C intake (mg/day)	1353	98 (59–163)	92 (52–163)	0.395
Zinc intake (mg/day)	1353	11 (7–16)	11 (7–17)	0.885
Iron intake (mg/day)	1353	12 (8–18)	12 (8–19)	0.432
Taking vitamin B12 supplement (%)	1493	3.7	0	0.166
Taking vitamin C supplement (%)	1499	4.5	1.6	0.520
Taking vitamin D supplement (%)	1497	4.5	0	0.108
Taking zinc supplement (%)	1499	2.4	3.2	0.664
Taking iron supplement (%)	1494	2.2	6.5	0.054
Serum vitamin B12 (pmol/L)	1492	333 (210–529)	320 (204–503)	0.522
Serum active B12 (pmol/L)	360	48 (29–81)	40 (21–77)	0.171
Serum iron (μmol/L)	1491	16 (11–22)	14 (11–19)	0.020
Serum ferritin (μmol/L)	1460	117 (44–317)	112 (41–308)	0.732
Serum transferrin (μg/L)	1492	33 ± 5	33 ± 6	0.339
Anaemia (%)	1498	10.4	29.0	< 0.001
Age at diagnosis of diabetes (years)	1493	56.0 ± 12.0	47.0 ± 16.1	< 0.001
Duration of diabetes (years at study entry)	1493	8.0 [2.1–15.0]	17.4 [10.8–24.9]	< 0.001
Diabetes treatment (%)	1498			< 0.001
Diet		25.6	4.8	
Oral agents ± non‐insulin injectables		54.2	40.3	
Insulin ± oral agents ± non‐insulin injectables		20.1	54.8	
Fasting glucose (mmol/L)	1492	7.1 [6.2–8.8]	8.9 [6.3–11.6]	0.001
HbA_1c_ (%)	1494	6.8 [6.2–7.7]	7.4 [6.6–9.8]	< 0.001
HbA_1c_ (mmol/mol)	1494	51 [44‐61]	57 [49‐84]	< 0.001
Height (cm)	1496	166 ± 10	168 ± 11	0.274
BMI (kg/m^2^)	1494	31.3 ± 6.2	31.1 ± 5.6	0.832
Central obesity (% by waist circumference)	1489	71.1	72.6	0.887
Supine SBP (mm Hg)	1496	145 ± 22	154 ± 31	0.034
Supine DBP (mm Hg)	1496	80 ± 12	82 ± 14	0.170
Pulse pressure (mm Hg)	1496	65 ± 18	72 ± 25	0.041
Heart rate (bpm)	1492	70 ± 12	76 ± 16	0.001
On antihypertensive medication (%)	1494	73.1	75.8	0.770
Total serum cholesterol (mmol/L)	1494	4.4 ± 1.1	4.5 ± 1.5	0.520
Serum HDL‐cholesterol (mmol/L)	1494	1.2 ± 0.3	1.2 ± 0.4	0.788
Serum triglycerides (mmol/L)	1494	1.5 (0.9–2.5)	1.7 (0.9–3.0)	0.165
On lipid‐modifying medication (%)	1494	67.9	69.4	0.890
On aspirin (%)	1492	37.6	30.6	0.286
Serum hsCRP (mg/L)	1492	2.5 (0.8–7.6)	3.6 (1.1–12.3)	0.009
Urinary albumin:creatinine (mg/mmol)	1474	3.1 (0.8–11.4)	10.5 (1.6–69.7)	< 0.001
eGFR categories (%)	1494			0.001
≥ 90 mL/min/1.73 m^2^		39.2	32.3	
60–89 mL/min/1.73 m^2^		45.0	33.9	
45–59 mL/min/1.73 m^2^		8.7	11.3	
30–44 mL/min/1.73 m^2^		4.6	11.3	
< 30 mL/min/1.73 m^2^		2.4	11.3	
Serum NTpro‐BNP (pg/mL)	1492	75 (18–313)	272 (41–1820)	< 0.001
Heart failure (%)	1499	6.0	17.7	0.002
Ischaemic heart disease (%)	1499	28.4	37.1	0.152
Atrial fibrillation (%)	1480	4.1	12.9	0.005
Cerebrovascular disease (%)	1499	7.9	21.0	0.001
Self‐reported claudication (%)	1499	8.6	24.2	< 0.001
Peripheral arterial disease (%)	1499	21.8	43.5	< 0.001
Distal symmetrical polyneuropathy (%)	1471	57.0	87.1	< 0.001
Diabetes‐related lower extremity amputation (%)	1499	0.3	11.3	< 0.001
Foot ulcer present at study entry (%)	1491	1.0	11.3	0.001
Prior hospitalisation for foot ulcer or foot ulcer present at study entry (%)	1499	1.8	25.8	< 0.001
Peripheral revascularisation/bypass (%)	1499	2.1	24.2	< 0.001
Any retinopathy (%)	1470	34.8	63.8	< 0.001
Depression (%)				
Any	1394	11.9	23.6	0.019
Major	1396	6.2	9.1	0.388

^a^ Small cell counts have been suppressed when there is the possibility of identification.

### 3.4 Determinants of First Incident Diabetes‐Related Foot Ulcer Hospitalisation

Independent determinants of (i) first DFU hospitalisation during follow‐up, and (ii) first ever DFU hospitalisation are shown in Table 4. Including all participants with type 2 diabetes, incident DFU hospitalisation was associated with male sex, marital status, age at diagnosis of diabetes, HbA_1c_, a history of DFU (its influence attenuating over time), a history of peripheral revascularisation/bypass, heart rate, and ln(NT‐proBNP) (its influence intensifying over time). Excluding the 44 with a history of DFU at study entry, except for a history of DFU (by design), marital status, and heart rate, with fasting serum glucose replacing HbA_1c_ as a marker of glycaemic control, the same variables entered the Cox model of time to first ever DFU hospitalisation, albeit with modified hazard ratios and no time dependence. The proportional hazards assumption was not violated. Vitamin C, iron and zinc intake (self‐reported supplements and estimated daily intake from dietary questionnaire) were not independently associated with incident DFU hospitalisation.

**TABLE 4:** Cox models of independent associates of (i) first incident diabetes‐related foot ulcer (DFU) hospitalisation in all FDS2 participants with type 2 diabetes (final model 60 events/1481 [98.8%] participants), and (ii) first ever incident DFU hospitalisation in FDS2 participants with type 2 diabetes with no DFU history (final model 44 events/1443 [99.2%] participants).

Outcome	Baseline variable	HR (95% CI)	*p*‐value
First DFU	Male	1.85 (1.08, 3.15)	0.025
	Married/de facto	0.37 (0.21, 0.64)	< 0.001
	Age at diabetes diagnosis (increase of 10 years)	0.67 (0.54, 0.83)	< 0.001
	HbA_1c_ (increase of 1% or 11 mmol/mol)	1.24 (1.07, 1.43)	0.004
	History of DFU	7.98 (3.43, 18.6)	< 0.001
	Peripheral revascularisation/bypass	5.27 (2.29, 12.1)	< 0.001
	Heart rate (increase of 1 bpm)	1.03 (1.01, 1.05)	0.008
	Ln (serum NT‐proBNP [pmol/L])^a^	1.58 (1.35, 1.86)	< 0.001
	*Time‐varying covariates*		
	History of DFU	0.48 (0.27, 0.85)	0.012
	Ln (serum NT‐proBNP [pmol/L])^a^	1.17 (1.05, 1.30)	0.003
First ever DFU	Male	2.21 (1.16, 4.20)	0.016
	Age at diabetes diagnosis (increase of 10 years)	0.59 (0.46, 0.75)	< 0.001
	Fasting serum glucose (increase of 1 mmol/L)	1.12 (1.05, 1.20)	0.001
	Peripheral revascularisation/bypass	5.20 (2.15, 12.6)	0.004
	Ln (NT‐proBNP [pmol/L])^a^	1.99 (1.67, 2.38)	< 0.001

^a^ A 2.72‐fold increase in NT‐proBNP corresponds to an increased risk of 1 in ln(NT‐proBNP).

## 4 Discussion

The present study found no association between micronutritional status or self‐reported intake of vitamin supplements and prevalent DFU or incident DFU hospitalisation in a community‐based cohort of people with type 2 diabetes. Associates of prevalent DFU and incident DFU hospitalisation included established risk factors such as DSPN and PAD. This is in contrast to the well documented high prevalence of inadequate dietary intake and/or deficiencies of micronutrients and minerals such as vitamin C, vitamin A, iron and zinc reported among patients with active DFU attending specialist multidisciplinary high risk foot clinics [12, 13, 30, 31].

There are a number of plausible explanations for these apparently contrasting findings. It is possible that nutritional status has a minor role in the development of a new DFU, with established risk factors such as DSPN, PAD and foot deformity being dominant in the pathogenesis of foot wounds [6, 32, 33]. The high prevalence of inadequate nutrition among patients attending specialist high risk foot clinics [12, 30] may indicate that this is a risk factor for more severe DFUs of longer duration with delayed healing in primary care or community settings. Clinic‐based patients with DFUs may thus represent a selected group in whom infection and comorbidities such as renal impairment may worsen nutritional status through anorexia and depression [34, 35] as part of a vicious cycle. It is possible that requirements of certain micronutrients are higher among people with diabetes and/or DFU, particularly those who smoke and/or have higher glucose levels with the corollary that standardised intake levels for the broader population may not be directly applicable to this high risk group [30, 36]. Additionally, a previous study has demonstrated only a moderate positive correlation between intake of micronutrients such as vitamin C determined by food frequency questionnaires and measured Vitamin C level [37], so it is possible our study design was unable to detect evidence of true micronutrient deficiencies using FFQ. There are small intervention studies suggesting a possible benefit of supplementation with micronutrients such as Vitamin C on DFU healing, however current guidelines do not recommend routine nutritional intervention for DFU healing, suggesting further evidence is required [13, 15, 38, 39]. Similarly, there is currently no evidence demonstrating nutritional interventions have a role in prevention of a new DFU.

We found a greater proportion of patients with anaemia among patients with prevalent DFU (29.5%) and incident DFU hospitalisation (29%), compared to those patients with no DFU (10.4%–10.7%). This may be secondary to associated factors such as more advanced chronic kidney disease and/or inflammation, as patients with prevalent DFU and incident DFU hospitalisation had higher uACR and lower eGFR than patients without DFU, and higher hsCRP was found in patients with incident DFU hospitalisation than those without DFU. High rates of anaemia (51.9%) have been described previously in a small study of patients with DFU attending a specialist high risk foot clinic, with functional iron deficiency detected in one quarter of patients (25.5%) [31]. We found no between‐group differences in serum ferritin or transferrin levels, however serum iron was lower in patients with prevalent DFU and/or incident DFU hospitalisation compared to patients with no DFU. While we did not further classify our participant group into those with absolute or functional iron deficiency, a low serum iron is seen in both of these iron deficient states [31]. Pena et al. found only 5.9% of patients with an active DFU had a low serum ferritin [12] which might reflect its role as an acute phase reactant thus complicating assessment of the adequacy of iron stores where there is inflammation and/or infection.

Vitamin C, iron and zinc intake (self‐reported supplements and estimated daily intake from FFQ) were not independently associated with either prevalent DFU or incident DFU hospitalisation in the present study. Independent associates of prevalent DFU included younger age at diabetes diagnosis, being married (protective), presence of retinopathy and DSPN, previous diabetes‐related amputation and peripheral revascularisation procedures. Independent associates of first ever incident DFU hospitalisation included being male, younger age at diabetes diagnosis, higher fasting serum glucose, history of peripheral revascularisation or bypass, and higher serum NT‐proBNP. These findings are consistent with the established understanding of the pathogenesis of a new DFU, with the most important factors being presence of DSPN, PAD and biomechanical factors as well as male sex, older age, longer diabetes duration and suboptimal glycaemic control being important contributors [32, 33]. This emphasises the importance of the established key principles of DFU management, with the provision of well‐coordinated, timely access to interdisciplinary care for people with active DFU required to achieve optimal outcomes [2, 40].

The present study had some limitations. Dietary intake of vitamin C, zinc and iron were determined via a self‐reported food frequency questionnaire. Biochemical measurement of indices of iron status were performed, but direct measurement of vitamin C and zinc levels was not possible as the specialised sample processing required could not be performed on stored frozen samples. The outcome of interest (incident DFU hospitalisation) was ascertained via data linkage and represents the severe end of the clinical spectrum—it is possible that nutritional status may have a more substantial role in the development of DFUs not requiring hospitalisation but this could not be ascertained in the present study. Full information regarding dose, frequency and duration of use of nutritional supplements was not always available [22] and may have provided important insights into the understanding of the relationship between the use of these supplements and development of DFU. The strengths of the present study are the large samples of participants followed for a long period, and its well characterised participants with type 2 diabetes.

In conclusion, we found no clear evidence of an association between dietary intake and/or supplements of vitamin C, iron or zinc and prevalent DFU or incident DFU hospitalisation. The strongest determinants of DFU prevalence and incident hospitalisation were established risk factors such as DSPN and PAD. Further research, such as prospective cohort studies examining the role of micronutrient intake and measured deficiency on incident DFU and high quality RCTs examining the effect of micronutrient supplementation on DFU healing outcomes, is required to improve our understanding of the role of nutrition in the development and healing of DFUs.

## Author Contributions

**Emma J. Hamilton:** conceptualization, data curation, methodology, investigation, supervision, writing – original draft. **Wendy A. Davis:** conceptualization, data curation, formal analysis, methodology, investigation, funding acquisition, supervision, writing – review and editing. **Ranita Siru:** data curation, investigation, writing – review and editing. **Mendel Baba:** data curation, investigation, writing – review and editing. **Timothy M. E. Davis:** conceptualization, data curation, methodology, funding acquisition, supervision, writing – review and editing.

## Funding

FDS2 was funded by the National Health and Medical Research Council (Project Grants 513781 and 1042231). This present activity has also been supported by the Western Australian Future Health Research and Innovation Fund (WA Near Miss Awards Emerging Leaders 2023‐24, Emma J. Hamilton).

## Ethics Statement

This research was approved by the Human Research Ethics Committee of the Southern Metropolitan Area Health Service (reference 07/397). All participants gave written informed consent prior to participation.

## Conflicts of Interest

The authors declare no conflicts of interest.

## Data Availability

Restrictions apply to the availability of data generated or analysed during this study to preserve patient confidentiality or because they were used under license. The corresponding author will on request detail the restrictions and any conditions under which access to some data may be provided.
